# Mesenchymal stem cells derived from human induced pluripotent stem cells improve the engraftment of myogenic cells by secreting urokinase-type plasminogen activator receptor (uPAR)

**DOI:** 10.1186/s13287-021-02594-1

**Published:** 2021-10-09

**Authors:** Ahmed Elhussieny, Ken’ichiro Nogami, Fusako Sakai-Takemura, Yusuke Maruyama, Natsumi Takemura, Wael Talaat Soliman, Shin’ichi Takeda, Yuko Miyagoe-Suzuki

**Affiliations:** 1 Department of Molecular Therapy, National Institute of Neuroscience, National Center of Neurology and Psychiatry, 4-1-1 Ogawa-higashi, Kodaira, Tokyo 187-8502 Japan; 2 Department of Neurology, Faculty of Medicine, Minia University, Minia, Egypt; 3 Department of Neurology, Neurological Institute, Graduate School of Medical Sciences, Kyushu University, Fukuoka, 812-8582 Japan; 4 Department of Gene Regulation, Faculty of Pharmaceutical Sciences, Tokyo University of Science, Noda, Chiba 278-8510 Japan

**Keywords:** Human iPS cells, Mesenchymal stem cells, Skeletal muscle, Muscle progenitors, Cell transplantation, Duchenne muscular dystrophy (DMD), Cytokine, Dystrophin, Urokinase-type plasminogen activator (uPA), uPA receptor (uPAR), Migration, Paracrine

## Abstract

**Background:**

Duchenne muscular dystrophy (DMD) is a severe X-linked recessive disease caused by mutations in the *dystrophin* gene. Transplantation of myogenic stem cells holds great promise for treating muscular dystrophies. However, poor engraftment of myogenic stem cells limits the therapeutic effects of cell therapy. Mesenchymal stem cells (MSCs) have been reported to secrete soluble factors necessary for skeletal muscle growth and regeneration.

**Methods:**

We induced MSC-like cells (iMSCs) from induced pluripotent stem cells (iPSCs) and examined the effects of iMSCs on the proliferation and differentiation of human myogenic cells and on the engraftment of human myogenic cells in the tibialis anterior (TA) muscle of *NSG-mdx*^*4Cv*^ mice, an immunodeficient dystrophin-deficient *DMD* model. We also examined the cytokines secreted by iMSCs and tested their effects on the engraftment of human myogenic cells.

**Results:**

iMSCs promoted the proliferation and differentiation of human myogenic cells to the same extent as bone marrow-derived (BM)-MSCs in coculture experiments. In cell transplantation experiments, iMSCs significantly improved the engraftment of human myogenic cells injected into the TA muscle of *NSG-mdx*^*4Cv*^ mice. Cytokine array analysis revealed that iMSCs produced insulin-like growth factor-binding protein 2 (IGFBP2), urokinase-type plasminogen activator receptor (uPAR), and brain-derived neurotrophic factor (BDNF) at higher levels than did BM-MSCs. We further found that uPAR stimulates the migration of human myogenic cells in vitro and promotes their engraftment into the TA muscles of immunodeficient *NOD/Scid* mice*.*

**Conclusions:**

Our results indicate that iMSCs are a new tool to improve the engraftment of myogenic progenitors in dystrophic muscle.

**Supplementary Information:**

The online version contains supplementary material available at 10.1186/s13287-021-02594-1.

## Introduction

Duchenne muscular dystrophy (DMD) is a devastating X-linked muscle disease that is caused by mutations in the DMD gene and affects 1 of 5000 male infants [1, 2]. Boys with DMD exhibit symptoms between 2 and 5 years of age, including delayed motor development, abnormal gait, and muscle weakness [2]. Progressive muscle degeneration leads to loss of ambulation when patients are 8–12 years old and cardio-respiratory failure when patients are in their 20 s and 30 s [2].

The *DMD* gene encodes the protein dystrophin. In myofibers, dystrophin is required for assembly of the dystrophin-glycoprotein complex (DGC) at the sarcolemma, and the DGC links the extracellular matrix (ECM) to the cytoskeleton [3]. Myofibers lacking dystrophin are easily damaged by contraction-induced mechanical stress, leading to repeated cycles of necrosis and regeneration of myofibers that result in chronic inflammation and gradual replacement of myofibers with fibrous and fatty tissues [4]. Transplantation of muscle stem/progenitor cells is a potential therapy for DMD, but the transplanted cells do not efficiently engraft and exert the therapeutic effects in the muscle of DMD patients [5].

In recent years, there has been considerable interest in the clinical application of mesenchymal stem cells (MSCs) for the treatment of muscle diseases. Several groups have demonstrated that transplantation of MSCs, through their paracrine functions, promotes the regeneration of skeletal muscle and ameliorates the dystrophic phenotype in animal models of DMD [6–13]. Therefore, we thought that MSCs might improve the engraftment of human myogenic progenitors by supporting their survival, proliferation, migration, and differentiation into myofibers in a paracrine manner.

MSC-like cells can be derived from human embryonic stem cells (hESCs) or human induced pluripotent stem cells (hiPSCs) [14–16]. hESC/hiPSC-derived MSCs are reported to have higher proliferative capacity and to show greater therapeutic effects than tissue-derived MSCs in several disease models [17–19]. Therefore, we utilized human hiPSCs to generate MSCs (iMSCs). Here, we report that iMSCs improved the engraftment of human myogenic cells in the TA muscle of immune-dystrophin-deficient *NSG*-*mdx*^*4Cv*^ mice. Our results also suggested that iMSCs increased the engraftment efficiency of myogenic cells by secreting uPAR. iMSCs may aid in the development of cell-based therapies to treat DMD.

## Results

### Derivation of MSCs from human iPS cells

**Fig. 1:**
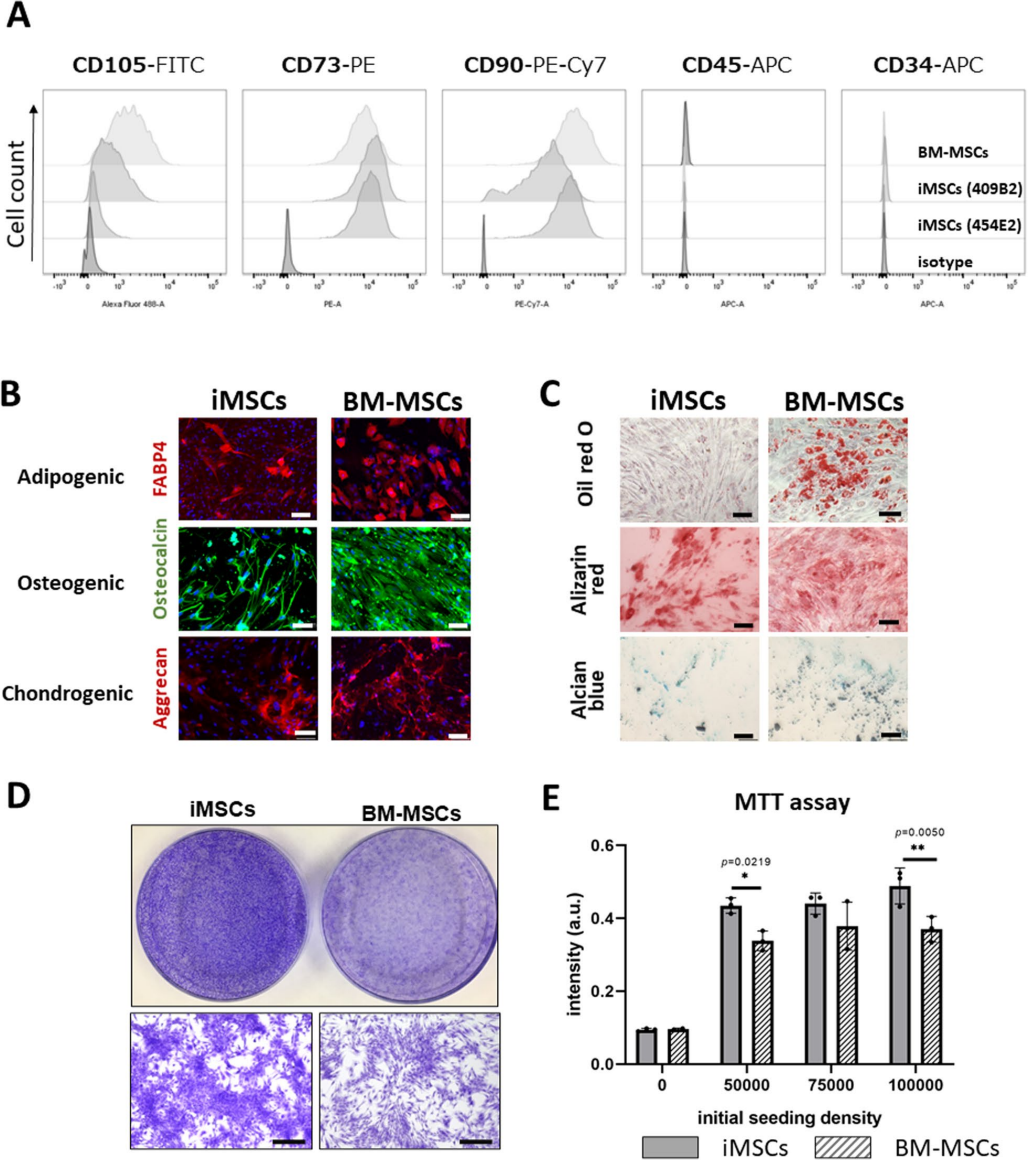
Mesenchymal stem cells induced from hiPSCs expressed MSC markers and retained the potential to differentiate into adipocytes, osteocytes, and chondrocytes in vitro. **A** Representative flow cytometry profiles of iMSCs (454E2), iMSCs (409B2), and BM-MSCs. iMSCs expressed the mesenchymal stem cell markers CD73 and CD105 but not the hematopoietic markers CD34 and CD45. Expression of CD105 was weak on iMSCs compared with BM-MSCs. **B** iMSCs and BM-MSCs were induced to differentiate into three mesenchymal lineages: adipocytes, osteocytes, and chondrocytes. Following differentiation, the cells were stained by immunocytochemistry for FABP4 (adipogenic marker, red), osteocalcin (osteogenic marker, green), or aggrecan (chondrogenic marker, red). Scale bar, 100 µm. **C** The cells were also stained with Oil red O to detect adipogenesis, Alizarin red to detect osteogenesis, and Alcian blue as an indicator of chondrogenesis. Scale bar, 100 µm. **D** iMSCs and BM-MSCs were seeded at a density of 1 × 10^5^ cells/100 mm petri dish in 10% FBS/DMEM. After 14 days of culture, the cells were stained with 0.05% crystal violet. **E** MTT assay of iMSCs and BM-MSCs. The experiments were repeated three times with the same tendency. Means ± SDs. Student’s *t-*test

We derived MSCs from three hiPSC cell lines, 201B7, 454E2, and 409B2, established from healthy donors [20, 21]. Induced MSCs (iMSCs) exhibited a fibroblastic spindle shape on plastic culture dishes (Additional file 1: Fig. 1A, B). Fluorescence-activated cell sorting (FACS) analysis showed that iMSCs expressed the mesenchymal stem cell markers CD73, and CD90, and were negative for the hematopoietic cell markers CD34 and CD45 (Fig. 1A). The expression of CD105 was constantly weaker in iMSCs than in BM-MSCs (Fig. 1A). Immunocytochemistry for FABP4, osteocalcin, and aggrecan indicated that the cells have the potential to differentiate into adipocytes, osteocytes, and chondrocytes under appropriate differentiation conditions (Fig. 1B). Positive staining with Alizarin red and Alcian blue in each differentiation condition further suggests the ability of iMSCs to differentiate into osteogenic and chondrogenic lineages, respectively (Fig. 1C). However, iMSCs differentiated into oil red O-positive adipogenic cells with much less efficiency than BM-MSCs. iMSC cultures were negative for the pluripotency markers OCT3/4, SOX2, and NANOG by immunocytochemistry and RT-qPCR, indicating that there were no residual pluripotent stem cells in the culture (Additional file 1: Fig. 1C, D). Thus, our iMSCs meet the minimal criteria for MSCs defined by the International Society for Cellular Therapy (ISCT) standards [22]. Next, we compared the proliferation of iMSCs to that of BM-MSCs. Crystal violet staining and MTT assays showed that the iMSCs had higher proliferation rates than BM-MSCs (Fig. 1D, E).

### iMSCs promoted proliferation and differentiation of human myogenic cells in vitro

**Fig. 2:**
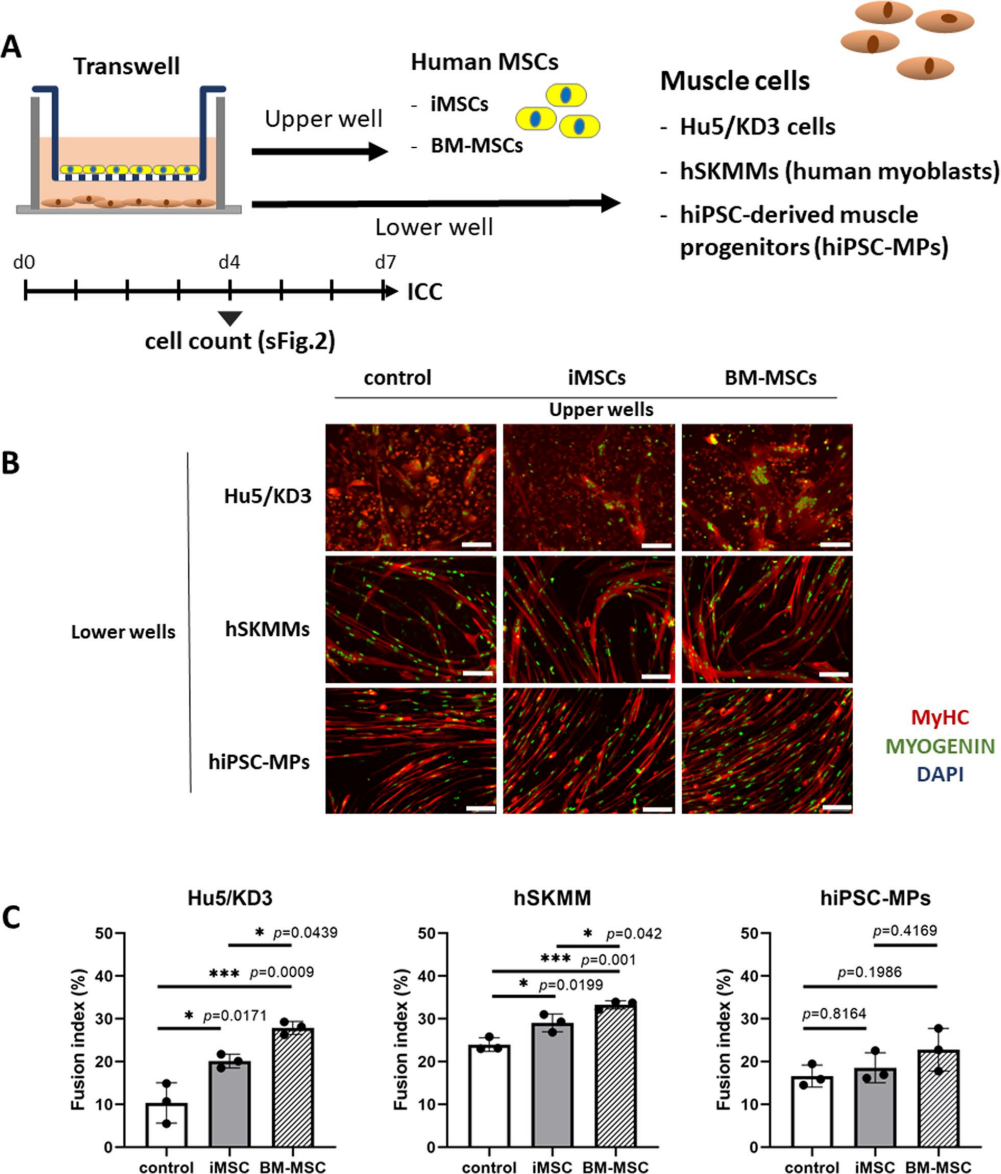
Human iMSCs and BM-MSCs promoted differentiation of human myogenic cells in a Transwell coculture system. **A** Experimental design. Human MSCs (iMSCs or BM-MSCs) (upper well) were cocultured with human myogenic cell lines: Hu5/KD3 cells, adult primary myoblasts (hSKMM), or hiPSC-derived muscle progenitors (lower well) in a Transwell system. **B** Representative images of myotubes formed by human myogenic cells after 7 days in culture. **C** Fusion index after 1 week of coculture with human MSCs. The cells were stained with MyHC (red), myogenin (green), and DAPI (nuclei, blue). The fusion index was calculated as the percentage of all nuclei that are myogenin-positive and within MyHC-positive myotubes. Scale bar, 100 μm. More than 30 fields from each sample (3 wells/group) were analyzed. Sidak’s analysis

Next, we examined the effects of MSCs on human myogenic cells by coculturing MSCs (either iMSCs or BM-MSCs) with human myogenic cells (Hu5/KD3 myoblasts, human primary skeletal muscle myoblasts (hSKMMs), or hiPSC-derived muscle progenitors) in a Transwell system (Fig. 2). In the Transwell system, MSCs and myogenic cells do not come into contact directly, allowing assessment of the effects of paracrine factors released from the MSCs on myogenic cells. Both iMSCs and BM-MSCs promoted the proliferation (Additional file 1: Fig. 2) of human myogenic cells (Hu5/KD3 cells). The effects on hiPSC-MPs were not statistically significant. The hSKMMs proliferated slowly, and their proliferation was not stimulated by coculture with MSCs. The higher fusion index at day 7 indicated that both iMSCs and BM-MSCs promoted the fusion of Hu5/KD3 cells and human skeletal myogenic cells (hSKMMs) at day 7. These results indicate that both iMSCs and BM-MSCs have similar abilities to promote the proliferation and differentiation of human myogenic cells in the Transwell coculture system (Fig. 2B, C).

### iMSCs improved engraftment of Hu5/KD3 myogenic cells in skeletal muscle of NSG-mdx^4Cv^ mice

**Fig. 3:**
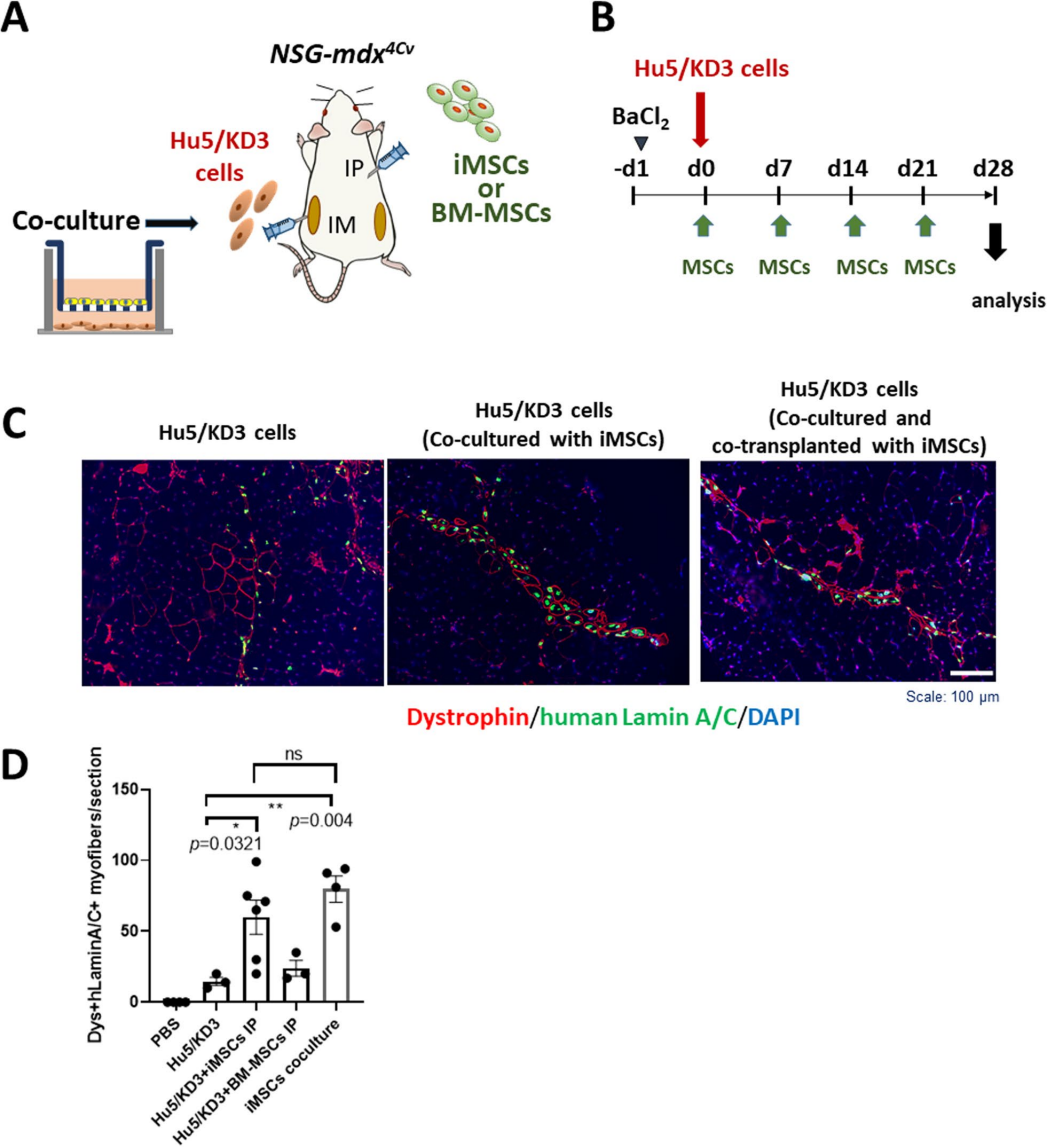
iMSCs promoted engraftment of Hu5/KD3 human myogenic cells in TA muscle of *NSG-mdx*^*4Cv*^ mice. **A** Experimental design of cotransplantation. Hu5/KD3 cells were cocultured with iMSCs or BM-MSCs in the Transwell system for 4 days before transplantation. The control group received only intramuscular injection of human myogenic Hu5/KD3 cells into the left TA muscle. The experimental groups received both intramuscular injection (IM) of Hu5/KD3 cells into the left TA muscle and intraperitoneal injection (IP) of BM-MSCs or iMSCs (with the same type of MSCs used for coculture). The right TA muscle in all groups was injected with 10% Matrigel/PBS (-). **B** Experimental schedule of transplantation. Fifty microliters of 1.2% BaCl_2_ was injected into the TA muscles on both sides 24 h (day 1) before transplantation of Hu5/KD3 cells to induce muscle regeneration. Hu5/KD3 cells were directly injected into the left TA muscle at day 0. MSCs were injected into the peritoneal cavity of the *NSG-mdx*^*4Cv*^ mice or *NOD/SCID* mice at days 0, 7, 14, and 21. The mice were killed at day 28. **C** Representative immunohistochemistry of cross sections of TA muscles of *NSG-mdx*^*4Cv*^ transplanted with Hu5/KD3 cells (left), Hu5/KD cells cocultured with iMSCs for 4 days (middle), and Hu5/KD3 cells cocultured with iMSCs and co-transplanted with iMSCs (IP)(right). The muscle sections were stained with anti-human lamin A/C (green), anti-dystrophin (red), and DAPI. Scale bar, 100 μm. **D** Numbers of human lamin A/C(+) dystrophin(+) myofibers/section of TA muscles of *NSG-mdx*^*4Cv*^ injected with Hu5/KD3 cells with or without MSCs. PBS: TA muscle injected with PBS. Hu5/KD3: TA muscle injected with Hu5/KD3 cells. Hu5/KD3 + iMSCs: Hu5/KD3 cells co-cultured and co-transplanted with iMSCs. Hu5KD3 + BM-MSCs: Hu5/KD3 cells co-cultured and co-transplanted with BM-MSCs. iMSCs coculture: Hu5/KD3 cells co-cultured with iMSCs and transplanted into TA muscle. Means ± SEMs. Tukey’s multiple comparisons test

Next, we examined whether MSCs support the engraftment of myogenic cells. A previous report showed that bone marrow-derived mesenchymal stromal cells injected into the peritoneal cavity ameliorated the pathology of *dystrophin/utrophin* double-knockout mice, a mouse model of severe DMD [8]. Therefore, to evaluate the paracrine effects of these cells, we first cocultured human myogenic cells with either BM-MSCs or iMSCs in a Transwell coculture system for four days, then transplanted MSCs intraperitoneally (IP) and transplanted the myogenic cells directly into the TA muscles of immuno-dystrophin-deficient *NSG-mdx*^*4CV*^ mice [23]. One day before the cell transplantation, both the right and left TA muscles were injected with 1.2% BaCl_2_ solution to damage the myofibers and induce muscle regeneration. We prepared five experimental groups for cell transplantation (Fig. 3). For the first group, 10% Matrigel in PBS was injected to the left TA muscle as a control. For the second group, Hu5/KD3 human myogenic cells were injected into the left TA muscle. For the third group, Hu5/KD3 myogenic cells were injected into the left TA muscle, and hiPSC-derived MSCs were transplanted intraperitoneally. For the fourth group, Hu5/KD3 cells were injected into the left TA, and BM-MSCs were injected intraperitoneally. For the fifth group, Hu5/KD3 cells were co-cultured with iMSCs for four days and then transplanted into the left TA muscle. For groups 3 and 4, MSCs were injected into the peritoneal cavity once per week for three weeks (4 IP injections in total) (Fig. 3B).

Four weeks after the transplantation of myogenic cells, the mice were killed, and the TA muscles were dissected and examined for engraftment of myogenic cells. Myofibers repaired by myogenic cells were detected by immunohistochemistry with dystrophin antibody and anti-human lamin A/C antibody. To exclude naturally occurring revertant dystrophin-positive fibers in *NSG-mdx*^*4Cv*^ mice [23], we counted dystrophin-positive (sarcolemma), and human lamin A/C-positive (nuclear membrane) myofibers (Fig. 3C, D). Intraperitoneal injection of iMSCs but not BM-MSCs increased the number of double-positive myofibers in TA muscles (Fig. 3C, D). The results were also confirmed by double staining with human spectrin (sarcolemma) and human lamin B1 staining (Additional file 1: Fig. 3A). No human lamin A/C-positive myofibers were found in the Matrigel-PBS-injected right TA muscles of any experimental group, indicating that MSCs injected into the peritoneal cavity did not migrate to the TA muscle and fuse to damaged myofibers there (Fig. 3C). Importantly, single transplantation of Hu5/KD3 cells, which had been co-cultured with iMSCs in Transwell plates, also showed numbers of double-positive myofibers comparable to co-transplantation with iMSCs (IP), suggesting that exposure to soluble factors from iMSCs was enough to improve the engraftment of human myogenic cells (Fig. 3D).

We also injected Hu5/KD3 myogenic cells into the TA muscles of immunodeficient *NOD/Scid* mice. The intraperitoneal injections of iMSCs also showed a tendency to promote engraftment of the Hu5/KD3 cells into the TA muscles of *NOD/Scid* mice, although this difference was not statistically significant (Additional file 1: Fig. 3B).

### Intraperitoneal transplantation of BM-MSCs and iMSCs reduced fibrosis of the diaphragm in NSG-mdx^4Cv^ mice

We also evaluated the effects of iMSCs on dystrophic pathology because MSCs have been reported to improve the dystrophic phenotype via paracrine effects throughout the body [6–13]. The grip test and treadmill test administered to *mdx* mice peritoneally injected with iMSCs showed a tendency of improvement of muscle function, but there was statistically no significant difference between control and treated *mdx* mice (Additional file 1: Fig. 4A, B). Hematoxylin and eosin (H.E.) staining showed no obvious improvement in the pathology of the right TA muscles, which were not injected with Hu5/KD3 cells, in *NSG-mdx*^*4Cv*^ mice or of *mdx* mice (data not shown). We compared the fiber cross-sectional area (CSA) in the right TA muscles in all groups; again, there was no significant difference in fiber sizes among all three groups (Additional file 1: Fig. 4A, B). Likewise, there was no significant difference in the CSA distribution of myofibers in the TA muscles between the treated and non-treated *mdx* mice (Additional file 1: Fig. 5C–E).

**Fig. 4:**
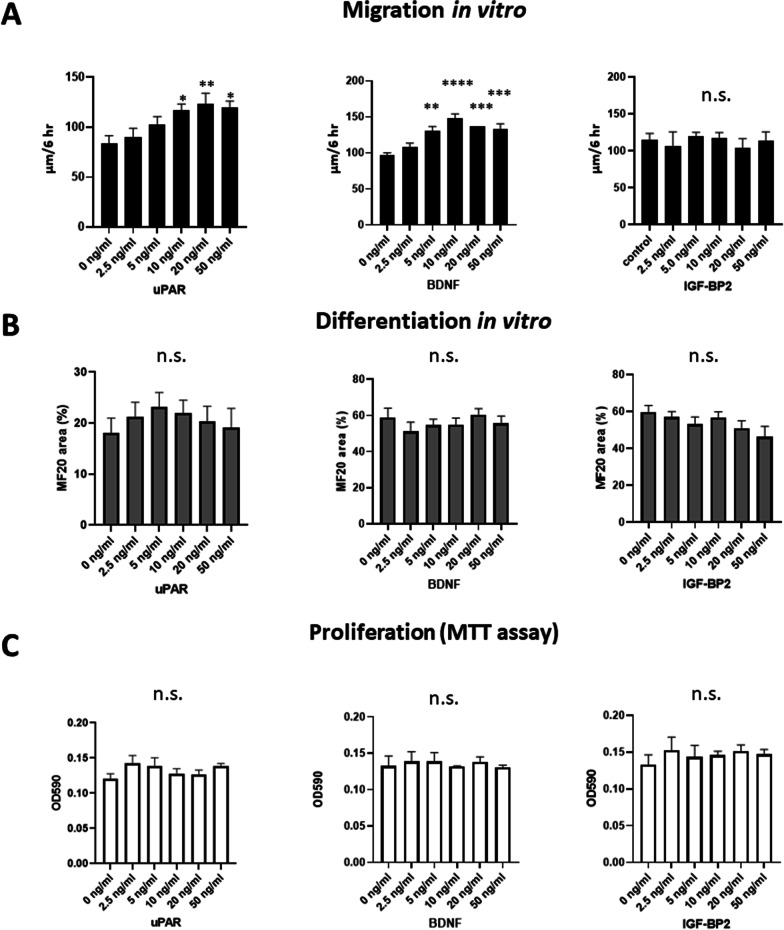
uPAR and BDNF stimulated migration of Hu5/KD3 cells in vitro. **A** Hu5/KD3 cells were seeded at a density of 1.0 × 10^6^/well in 6-well collagen plates in the presence of uPAR (left), BDNF (middle), or IGFBP2 (light) (0–50 ng/ml). The next day, a straight scratch was made with a 200 μl pipette tip (0 h) and photographed under an inverted microscope. Six hours later, the cells were again photographed (Additional file 1: Fig. 6). Six views/condition were recorded. Migration activity was evaluated by measuring the width of the wound. **B** Hu5/KD3 cells were plated onto 24-well collagen plates at 2 × 10^5^ cells/well and induced to differentiate in 10% FBS/DMEM medium supplemented with recombinant human uPAR (left), BDNF (middle) or human IGFBP2 protein (right) (0–50 ng/ml). Four (uPAR) or five days (BDNF, IGFBP2) later, the cells were fixed and stained with an anti-muscle myosin heavy chain antibody (MF20) and DAPI (nuclei). Shown are averages of 4 wells/condition. The percentage of MF20-positive area was quantified using ImageJ software. **C** Hu5/KD3 cells were plated onto 24-well collagen plates at 1 × 10^4^ cells/well in 10% FBS/DMEM supplemented with recombinant human uPAR (left), BDNF (middle), or IGFBP2 (right) protein (0–50 ng/ml). Three days later, an MTT assay was performed (4 wells/condition). In (**A**–**C**), data are shown as the mean ± SEM. Dunnett’s analysis. n.s., not significant. **, *p* < 0.01. ***, *p* < 0.001. ****, *p* < 0.0001

**Fig. 5:**
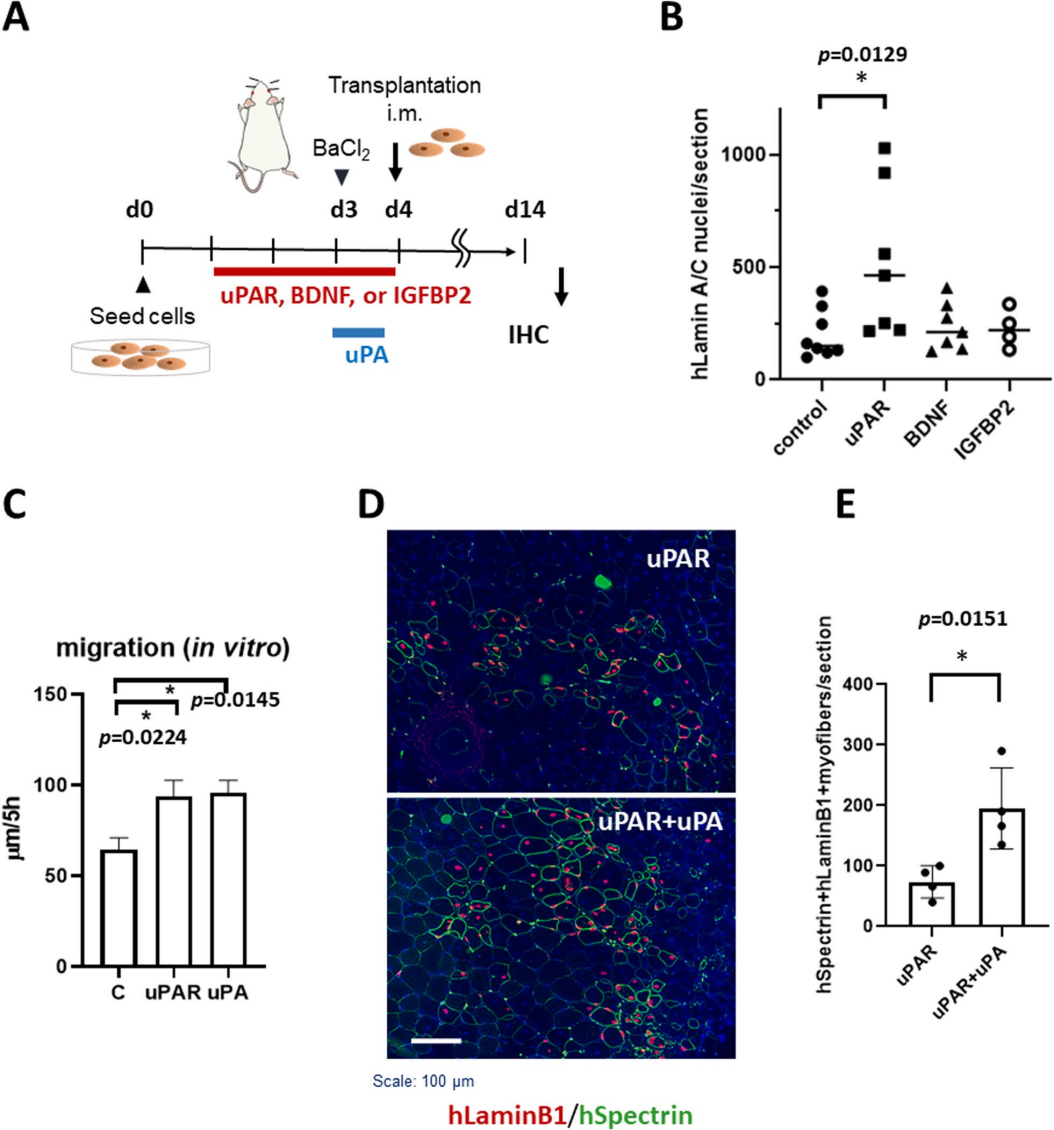
Recombinant human uPAR protein promoted engraftment of human myogenic cells in TA muscle of *NOD/Scid* mice. **A** Outline of the transplantation experiments. Before transplantation, myogenic cells were cultured in 10% FBS/DMEM supplemented with 20 ng/ml uPAR, BDNF, or IGFBP2 for 3 d. Recombinant uPA was added 24 h before transplantation. The TA muscles of NOD/Scid mice were injured with 1.2% BaCl_2_ solution 24 h before cell transplantation and injected with 1.5 × 10^6^ cells (Hu5/KD3 cells) or 1.0 × 10^6^ cells (hiPSC-derived muscle progenitors) in 50 μl of PBS containing 5 μg/ml uPAR or BDNF. Two weeks after transplantation, the mice were killed, and the TA muscles were dissected for immunohistochemical analysis using an anti-human lamin A/C antibody and human spectrin (green). **B** Numbers of human lamin A/C-positive nuclei/section of TA muscle injected with Hu5/KD3 cells treated with the indicated cytokines. *n* = 4–8 mice/group. Data are shown as the means ± SEMs. Dunnett’s analysis. n.s., not significant. *, *p* < 0.05. **C** Hu5/KD3 cells were seeded at a density of 1.0 × 10^6^/well in 6-well collagen plates in the absence or presence of uPAR (10 ng/ml) or uPA (10 ng/ml). The next day, a straight scratch was made with a 200 μl pipette tip (0 h). Migration activity in 5 h was evaluated by measuring the width of the wound. Eight points/condition. Data are shown as the means ± SEMs. Dunnett’s analysis. *, *p* < 0.05. **D** Representative immunostaining of cross sections of TA muscles injected with Hu5/KD3 cells with uPAR (5 µg/ml) or uPAR + uPA (5 µg/ml, each). Cells were cultured in 10% FBS/DMEM supplemented with 20 ng/ml uPAR or 20 ng/ml uPAR + 20 ng/ml uPA for 3 d. Myofibers formed by Hu5/KD3 cells were identified with anti-human lamin B1 antibody (red) and human spectrin (green). **E** Quantitative analysis of (**D**). *n* = 4 mice/group. Unpaired two-tailed Student’s *t*-test

*Mdx* mice generally show a milder dystrophic muscle phenotype than DMD patients; however, the diaphragm of *mdx* mice shows progressive loss of myofibers and severe fibrosis. Therefore, we evaluated the fibrosis of the diaphragm in *NSG*-*mdx*^*4CV*^ mice with Sirius red staining. Surprisingly, the fibrous areas in the diaphragms of mice that received intraperitoneal injections of either iMSCs or BM-MSCs were significantly reduced compared to those of controls (Additional file 1: Fig. 4C, D). The Sirius red-stained area in the TA muscles was also examined; however, there was no significant difference between the iMSC- or BM-MSC-injected groups and the controls (data not shown).

### iMSCs and BM-MSCs produce different cytokines

To understand the mechanisms by which iMSCs improved engraftment of myogenic cells, we examined the cytokines secreted by iMSCs or BM-MSCs into the culture medium using a membrane-based human cytokine antibody array containing 120 targets. BM-MSCs and iMSCs strongly expressed many shared cytokines (TIMP-2, MCP-1, IL-8, TIMP-1, SDF-1a, IGFBP6, and TNFR1). However, iMSCs showed higher expression of IGFBP2, uPAR, and BDNF than BM-MSCs. BM-MSCs expressed osteoprotegerin (OPG) and IL-6 at higher levels than iMSCs (Table 1, Additional file 1: Fig. 6).

**Table 1:** Relative levels of cytokines in conditioned medium of BM-MSCs and iMSCs analyzed by cytokine array analysis

	BM-MSCs-1		BM-MSCs-2		iMSCs (409B2)-1		iMSCs (409B2)-2		iMSCs (201B7)	
	Cytokine	Signal intensity (arbitrary unit: a.u.)	Cytokine	Signal intensity (a.u.)	Cytokine	Signal intensity (a.u.)	Cytokine	Signal intensity (a.u.)	Cytokine	Signal intensity (a.u.)
1	TIMP-2	641	TIMP-2	378	TIMP-2	341	TIMP-2	351	TIMP-2	462
2	**OPG**	470	MCP-1	290	IL-8	279	MCP-1	195	TIMP-1	279
3	**IL-6**	364	**OPG**	289	MCP-1	203	IGFBP6	151	IL-8	251
4	MCP-1	357	IGFBP6	214	TIMP-1	136	TIMP-1	150	**IGFBP2**	240
5	IL-8	229	**IL-6**	214	**IGFBP-2**	83	**IGFBP2**	138	MCP-1	239
6	TIMP-1	224	IL-8	152	**uPAR**	63	IL-8	100	IGFBP6	171
7	IGFBP6	201	TIMP-1	143	sTNFRI	44	**uPAR**	85	**uPAR**	151
8	sTNFRI	99	LIGHT	97	**BDNF**	35	SDF-1alpha	55	**BDNF**	98
9	VEGF	66	sTNFR1	81	SDF-1alpha	33	**BDNF**	37	sTNFRI	97
10	SDF-1 alpha	52	SDF-1 alpha	66	ANG	28	ANG	42	IGFBP4	60

The cytokines highly upregulated in BM-MSCs (OPG. IL-6) or in iMSCs (IGFBP-2, uPAR and BDNF) are in bold

**Fig. 6:**
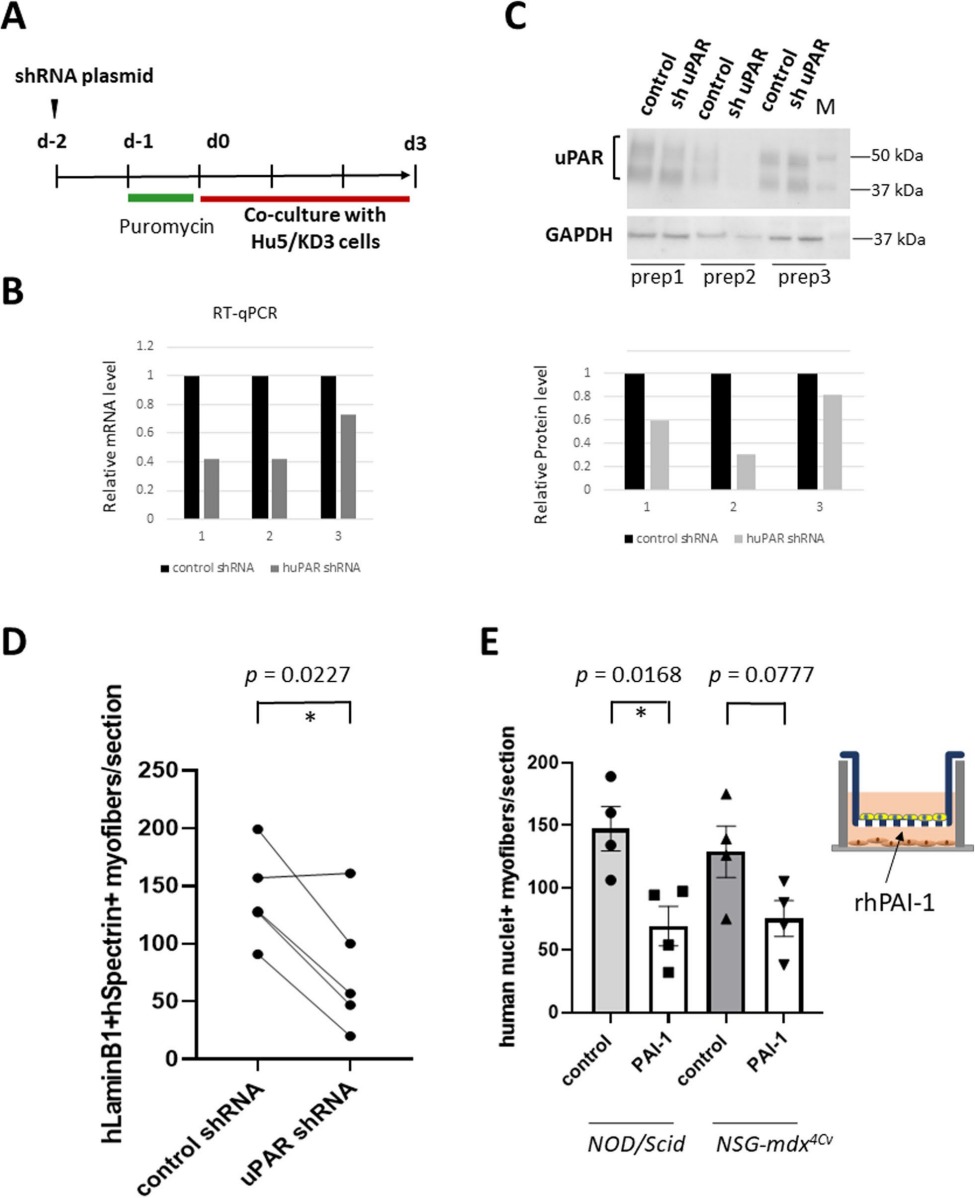
Knockdown of uPAR in iMSCs reduced the efficiency of cell transplantation. **A** Outline of the experiments. iMSCs were transfected with huPAR shRNA plasmids or control shRNA plasmids. After puromycin treatment for 24 h, iMSCs were co-cultured with Hu5/KD3 cells in 10% FBS/DMEM in a Transwell plate for 3 d. The TA muscles of NOD/Scid mice were injured with 1.2% BaCl_2_ solution 24 h before cell transplantation and injected with 5 × 10^5^ myogenic cells (Hu5/KD3 cells). Cells co-cultured with iMSCs transfected with control shRNA plasmids were injected into right TAs (control), and cells co-cultured with iMSCs transfected with uPAR shRNA plasmids were injected left TAs. Ten days after transplantation, the mice were killed, and the TA muscles were dissected for immunohistochemical analysis using an anti-human lamin B1 antibody and human spectrin antibody as described in Fig. 5. **B** RT-qPCR analysis performed on RNAs extracted from iMSCs transfected with shRNA plasmids. Prep. 1 and Prep. 2 showed 60% reduction of uPAR mRNA. **C** Western blotting was performed using proteins extracted from iMSCs transfected with uPAR shRNA plasmids or control shRNA plasmids. Prep. 1 and Prep. 2 showed 40–70% reduction of uPAR protein. **D** Numbers of human lamin B1-positive, human spectrin-positive myofibers in TA muscles transplanted with Hu5/KD3 cells after co-culture with iMSCs (summary of two independent experiments). *n* = 5 mice. Paired *t*-test. **E** Recombinant PAI-1 (100 ng/ml) was added to the coculture and three days later Hu5/KD3 cells were transplanted into left TA muscles of NOD/Scid mice (*n* = 4) or to NSG-mdx^4Cv^ mice (*n* = 4). Right TA muscles were injected with Hu5/KD3 cells that had been co-cultured with iMSCs without PAI-1 (control). Data are shown as the mean ± SEM

### uPAR stimulated migration of human myogenic cells in vitro and improved their engraftment in TA muscle of NOD/Scid mice

Finally, we tested whether uPAR, BDNF, or IGFBP2 promotes the engraftment of myogenic cells into immunodeficient *NOD/Scid* mice. Recombinant human uPAR and BDNF but not IGFBP2 protein stimulated the migration of human myogenic cells in vitro (Fig. 4A, Additional file 1: Fig. 6), while the proliferation of the cells (MTT assay) and differentiation (muscle myosin heavy chain staining) of the cells were not improved by uPAR, BDNF, or IGFBP2 (Fig. 4B, C). To examine the effects of uPAR, BDNF, or IGFBP2 on the engraftment of transplanted myogenic cells, we cultured Hu5/KD3 cells in 10% FBS/DMEM supplemented with uPAR, BDNF, or IGFBP2 at a concentration of 20 ng/ml for 3 days, suspended the cells in a buffer containing 5 μg/ml recombinant uPAR or BDNF and 5% Matrigel, and injected the suspension into the TA muscles of *NOD/Scid* mice. Immunohistochemical analysis using human lamin A/C antibody and human spectrin antibody revealed that only uPAR significantly improved the engraftment of Hu5/KD3 cells in the TA muscle of *NOD/Scid* mice (Fig. 5A, B). To clarify the mechanisms by which uPAR improved the engraftment of myogenic cells, we tested whether urokinase-type plasminogen activator (uPA) enhanced the effects of uPAR on cell engraftment. Interestingly, recombinant uPA stimulated migration of myogenic cells (Fig. 5C) and further increased the number of human lamin B1-positive, human spectrin-positive myofibers in the host muscle (Fig. 5D, E).

Finally, we knocked down uPAR expression in 454E2-hiPSC-derived MSCs using shRNA plasmids (Fig. 6). A cocktail of three plasmids reduced the expression of uPAR 30–60% at the protein level (RT-qPCR) and 20–70% at the mRNA level (Western blotting) (Fig. 6). The knockdown of uPAR in iMSCs reduced the efficiency of cell transplantation, suggesting that iMSC-derived uPAR plays a major role in promoting the engraftment efficiency of myogenic cells.

To understand the mechanisms by which uPAR improved cell engraftment, we added a recombinant human plasminogen activator inhibitor-1 (PAI-1), a natural inhibitor of uPA, to the co-culture medium (100 ng/ml). As expected, PAI-1 reduced the efficiency of cell transplantation (Fig. 6E). This observation supports our hypothesis that iMSCs improved cell engraftment through activation of uPA by uPAR.

## Discussion

Transplantation of muscle stem/progenitor cells is a potential approach to treat DMD, but it faces many challenges. One factor that inhibits successful transplantation of muscle stem/progenitor cells is the hostile microenvironment caused by pathological changes in the DMD muscle [12, 13]. MSCs are unique multipotent stem cells in that they possess not only multipotent differentiation ability but also have paracrine functions that support muscle regeneration by promoting the activation, proliferation, and differentiation of muscle stem/progenitor cells as well as modulating immune responses [6–13]. However, MSCs isolated from tissues have limited proliferative potential for expansion in culture while maintaining their regenerative function [24]. In contrast, MSCs induced from human pluripotent stem cells were reported to expand for up to 40 passages (120 population doublings) without loss of plasticity or replicative senescence [26]. Therefore, we induced iMSCs from hiPSCs and tested whether these iMSCs improve the engraftment of human myogenic cells transplanted into *mdx* muscle.

iMSCs exhibited a fibroblast-like spindle shape on plastic dishes, expressed the MSC markers CD73, CD90, and CD105, and differentiated into three mesenchymal lineages: osteogenic, adipogenic, and chondrogenic cells. The higher proliferation activity and poorer adipogenic differentiation of iMSCs than BM-MSCs are in agreement with recent reports [25, 26].

Our coculture experiments using a Transwell system showed that both iMSCs and BM-MSCs stimulated the proliferation of myogenic cells and their differentiation to similar extents. Previous reports have shown that MSCs secrete many kinds of cytokines [27, 28]. Our results also indicated that iMSCs and BM-MSCs secrete a variety of cytokines, growth factors, and chemokines (Table 1, Additional file 1: Fig. 6). Therefore, it is likely that such soluble factors promoted the proliferation and differentiation of human myogenic cells in our coculture system.

Intraperitoneal injection of iMSCs or BM-MSCs significantly reduced fibrosis in the diaphragm in *NSG-mdx*^*4Cv*^ and *mdx* mice. We identified several cytokines, such as TIMP1, TIMP2, IL-8, SDF-1a, MCP-1, IGFBP6, and sTNFR1, as candidate factors contributing to the reduction of fibrosis in the diaphragm of *NSG-mdx*^*4Cv*^ and *mdx* mice. TIMP1/2, natural inhibitors of matrix metalloproteinases (MMPs), are good candidates because upregulation of MMP9 is reported to promote fibrosis in muscular dystrophy [29].

iMSCs promoted the engraftment of human myoblasts (Hu5/KD3 cells) in the TA muscle of *NSG-mdx*^*4Cv*^ mice. We cocultured Hu5/KD3 cells with iMSCs for four days before transplantation. Because co-culture with iMSCs enhanced the engraftment of myogenic cells, we think that exposure to soluble factors from iMSCs was critical for improving cell engraftment.

To identify engraftment-promoting factors, we examined the cytokines secreted by BM-MSCs and iMSCs by using a cytokine array. Although BM-MSCs and iMSCs showed similar cytokine expression patterns, iMSCs expressed uPAR, BDNF, and IGFBP2 at higher levels than BM-MSCs. To determine which cytokines promoted engraftment of the cells, we tested the effects of recombinant cytokines on the proliferation, differentiation, and migration of human myogenic cells in vitro. Interestingly, recombinant uPAR and BDNF significantly increased the motility of Hu5/KD3 cells on collagen-coated culture plates. In contrast, the effects of these factors on cell proliferation or differentiation were not evident in our in vitro system.

Although both uPAR and BDNF stimulated the migration of Hu5/KD3 cells in vitro, only uPAR promoted the engraftment of myogenic cells into the TA muscles of *NOD/Scid* mice (Fig. 5). Interestingly, recombinant uPA also stimulated migration of myogenic cells in a wound-healing assay in vitro and further enhanced the effects of uPAR in vivo*.* uPA and its receptor uPAR initiate a series of proteolytic cascades that degrade components of the extracellular matrix and enhance the migration of a variety of cells [30]. Migrating, undifferentiated myoblasts have also been reported to express uPA and uPAR. Furthermore, previous studies demonstrated that uPA/uPAR facilitates the proliferation, migration, and fusion of muscle satellite cells or myoblasts [31–35].

Our co-culture experiments, however, suggested that iMSC-derived soluble factors acted directly on human myogenic cells rather than acting on the microenvironments of the host muscle. In addition, human and mouse uPAR share approximately 60% amino acid sequence identity and the receptor-ligand interaction is highly species-specific [36], suggesting that iMSC-derived uPAR interacted with human uPA and potentiated Hu5/KD3 myogenic cells in co-culture to survive and regenerate myofibers in the host muscle. In clinical settings, however, human uPAR would activate endogenous uPA and further promote engraftment of myogenic cells.

## Conclusions

We induced MSC-like cells from human iPSCs (iMSCs). iMSCs promoted the engraftment of human myogenic cells in the TA muscles of *NSG-mdx*^*4Cv*^ mice. Our results suggested that iMSCs promoted the engraftment of human myogenic cells via secretion of uPAR. iMSCs, which possess properties distinct from those of BM-MSCs, are a promising new tool for cell therapies to treat Duchenne muscular dystrophy.

## Materials and methods

### Induction of myogenic cells from hiPSCs

201B7, 409B2, and 454E2 iPSCs were provided by S. Yamanaka at Kyoto University. 201B7 was generated from a healthy donor using retroviral vectors [20]. 409B2 and 454E2 were integration-free hiPS clones generated from healthy donors using episomal vectors [21]. hiPSCs were cultured on iMatrix-511 (Nippi)-coated 6-well plates in StemFit AK02N medium (Ajinomoto) supplemented with penicillin/streptomycin/amphotericin B (1% v/v) (FujiFilm) as described previously [37, 38]. Myogenic cells were induced from hiPSCs as described [37, 38]. After floating culture, cells were induced to differentiate into myogenic cells on collagen type I-coated 10-cm dishes (Iwaki) in 10% FBS (Gibco)/DMEM (FujiFilm) in the presence of 1 μM SB431542 (Wako) for one week. The induced cells were then collected and incubated with ERBB3-APC (1B4C3, BioLegend), CD57(HNK-1)-PE (clone TB03, Miltenyi Biotec), and CD271-BB515 (C40-1457, BD Pharmingen). Myogenic cells were sorted as the CD57(-) CD271(+) ERBB3(+) fraction [37, 38].

### Derivation of mesenchymal stem cells from hiPSCs (iMSCs)

MSCs were induced from hiPSCs using the STEMdiff Mesenchymal Progenitor kit (Stemcell Technologies) according to the manufacturer’s protocol. In brief, hiPSCs were first induced into early mesoderm progenitor cells with STEMdiff Mesenchymal Induction Medium. Four days later the medium was changed to MesenCult-ACF medium. The cells were then passaged into 6-well plates coated with MesenCult-ACF Attachment Substrate. At day 24, the induced cells were analyzed for cell surface markers, proliferative potential, and differentiation potential. In this study, iMSCs were used between passages 4 and 8.

### Human bone marrow MSCs (BM-MSCs)

BM-MSCs were purchased from Lonza (PT-2501) and cultured on gelatin-coated 6-well plates (Iwaki) using MSCGM Mesenchymal Stem Cell Growth Medium BulletKit (Lonza). Cells were used between passages 4 and 8.

### shRNA knockdown experiments

To reduce uPAR expression in iMSCs, we transfected 454E2-iMSCs with uPAR shRNA plasmids (human) (Santa Cruz sc-36781-SH) or control shRNA plasmids (human) (sc-108060) using FuGENE HD (Promega). For selection of transfected cells, puromycin (Millipore) was added to the culture medium at a concentration of 1 ug/ml for 24 h. Knockdown efficiency was evaluated by RT-qPCR and Western blotting as described [37, 38]. For Western blotting, a mouse monoclonal antibody against full length human uPAR protein (E-3, Santa Cruz) was used. After incubation with a second antibody and ECL reaction, the signal was recorded and quantitated with BIO-RAD ChemiDoc MP imaging system.

### Human myogenic cells

Hu5/KD3 cells are a human myoblast cell line [39]. The cells were cultured on collagen-coated dishes (Iwaki) in high-glucose DMEM (FujiFilm) supplemented with 20% FBS (Gibco) and 2% Ultroser G (Biosepta, Pall) as previously described [39]. Adult human skeletal muscle myoblasts (hSKMM) were obtained from Lonza (#CC-2580) and cultured on collagen-coated dishes (Iwaki) in 10% FBS/high glucose DMEM. Recombinant human uPAR protein (R&D Systems), recombinant BDNF (Peprotech), or recombinant IGF-BP2 (Peprotech) was added to the culture at different concentrations (2.5, 5.0, 10, 20, or 50 ng/ml).

### MTT assay

The cells were plated in 24-well collagen-coated plates at different cell densities: 5 × 10^4^, 7.5 × 10^4^, and 1.0 × 10^5^ cells per well (*n* = 3 for each group). The next day, 0.1 ml of 0.5% MTT solution was added per well and incubated for 3 h in a CO_2_ incubator. After aspiration of the medium, 1.0 ml of acid isopropanol was added to each well, and then the well contents were transferred to 1 ml tubes and centrifuged. The absorbance intensity of the supernatant at OD_590_ nm was measured in a plate reader (BioTek).

### Wound healing migration assay

Hu5/KD3 cells were seeded at a density of 1.0 × 10^6^ cells/well in 6-well collagen-coated plates (Iwaki) in 10% FBS/DMEM supplemented with cytokines at different concentrations (0–50 ng/ml). The next day, a straight scratch was made in individual wells with a sterile 200 μl pipette tip and photographed with an Olympus DP21 attached to an Olympus CKX41 inverted light microscope. After 6 h of culture in a CO_2_ incubator, the cells were again photographed (6 views/condition), and the gaps were measured with ImageJ software.

### Trilineage differentiation of MSCs

The trilineage differentiation potential of induced MSCs was examined using a human mesenchymal stem cell functional identification kit (R & D Systems) according to the manufacturer’s protocols.

#### Osteogenic differentiation

Cells were seeded at a density of 4.2 × 10^3^ cells/cm^2^ in αMEM basal medium in collagen-coated 24-well plates, and were cultured in a 5% CO_2_ incubator at 37 °C. When the cells reached 50–70% confluency, the medium was replaced with osteogenic differentiation medium. After 21 d, the cells were fixed for immunostaining or Alizarin red S staining (Sigma).

#### Adipogenic differentiation

Cells were seeded at a density of 2.1 × 10^4^ cells/cm^2^ in αMEM basal medium in collagen-coated 24-well plates and cultured in a 5% CO_2_ incubator at 37 °C. When the cells had grown to 100% confluence, the medium was replaced with 0.5 mL of adipogenic differentiation medium. After 21 d, the cells were fixed for immunostaining or Oil red O staining (Sigma). Nuclei were counterstained with Harris’ hematoxylin.

#### Chondrogenic differentiation

Cells (2.5 × 10^5^) were centrifuged at 200 × g for 5 min at RT in a 15-mL conical tube and resuspended in 1.0 mL of D-MEM/F-12 basal medium. The cells were again centrifuged, resuspended in 0.5 mL of chondrogenic differentiation medium, and centrifuged to form a cell pellet. The pellets were incubated upright in a 5% CO_2_ incubator at 37 °C. After 21 d, the chondrocyte pellets were fixed and sectioned.

### Immunocytochemistry

Cells were fixed in 4% paraformaldehyde for 10 min and treated with 0.1% Triton X-100 for 10 min at RT. After blocking with 5% goat serum (Cedarlane)/2% bovine serum albumin (BSA; Sigma) in PBS, the cells were reacted with primary antibodies: a rabbit polyclonal myogenin antibody (Santa Cruz Biotechnology) and an anti-pan myosin heavy chain (MHC) antibody, MF20 (R&D Systems) at a 1:200 dilution overnight at 4 °C. To detect the differentiation of MSCs, an anti-human osteocalcin antibody (R&D Systems, #967801), an anti-mouse FABP4 antibody (R&D Systems, #967799), or an anti-human aggrecan antibody (R&D Systems, #967800) was used. For detection of undifferentiated human iPS cells, an anti-hSOX2 antibody (Cell Signaling, #3579), anti-hNANOG antibody (R&D Systems, AF1997), or an anti-hOCT-3/4 antibody (R&D Systems, AF1759) was used. The next day, after washing with PBS (−), the cells were incubated with Alexa 568 goat anti-mouse IgG2b, Alexa 488 goat anti-rabbit IgG, Alexa 568 donkey anti-goat IgG, or Alexa 488 donkey anti-mouse IgG (Molecular Probes) for 2 h. The nuclei were then stained with DAPI (Tokyo Chemical Industry). The images were recorded with an all-in-one microscope (BZ-X810) and analyzed with hybrid cell count software (Keyence).

### RNA isolation, cDNA synthesis, and qPCR

RT-qPCR was performed as previously described [37, 38]. RNA was isolated from cells with TRIzol (Invitrogen) or an RNeasy Mini Kit (Qiagen) and reverse-transcribed into cDNA using a PrimeScript RT reagent kit (Perfect Real Time, Takara). cDNA was amplified with SYBR Premix EX Taq II (Til RNaseH Plus, Takara) and the primer sets listed in Additional file 1: Table 1. The signal was monitored with a CFX Connect Real-Time System (Bio-Rad), and ΔCt (1/2^ (Cq of the gene-median Cq)) was calculated. The glyceraldehyde-3-phosphate dehydrogenase (GAPDH) signal was used for normalization.

### FACS analysis

Cells were incubated with antibodies diluted as suggested by the suppliers in 200 μl of PBS containing 2% FBS (wash buffer) for 30 min on ice, washed in wash buffer, and analyzed using a FACSAria Fusion cell sorter (BD Biosciences). For analysis of iMSCs, CD73-PE (clone AD2, BD Pharmingen), CD105-FITC (clone 266, BD Pharmingen), CD90-PE-Cy7 (clone 5E10, BD Pharmingen), CD34-APC (clone 581, BD Pharmingen), and CD45-APC (clone HI30, BD Pharmingen) were used. The results were analyzed using BD FlowJo software (v.10).

### Coculture in a transwell system

MSCs were plated on Transwell inserts (3.0-µm pore polycarbonate; Corning). These inserts were placed in collagen-coated 12-well plates (Iwaki) already containing cultured human myogenic cells at a ratio of 1:1 for 4 d or 1 wk using 10% FBS/DMEM. To evaluate cell proliferation, nuclei were stained with DAPI and counted using Keyence hybrid cell count software. For evaluation of differentiation, the fusion index was calculated: myogenin-positive nuclei inside the myotubes/total nuclei × 100%. Recombinant human plasminogen activator inhibitor (PAI-1) (Sigma-Aldrich) was added to the culture medium at 100 ng/ml.

### Mice and cell cotransplantation

*NSG-mdx*^*4cv*^ mice [23] were kindly provided by M. Kyba of the University of Minnesota. C57BL/6-background *mdx* mice were a generous gift from T. Sasaoka (Niigata University, Japan). *NOD/Scid* mice (NOD/ShiJic-*scid*Jcl), which lack T- and B-cell function and have decreased natural killer (NK) cell activity, complement activity, and macrophage function, were purchased from CLEA Japan, Inc.

Under general anesthesia with isoflurane, both right and left TA muscles of *NSG-mdx*^*4Cv*^, *mdx*, or *NOD/Scid* mice were injured by direct injection of a solution of 1.2% BaCl_2_ in PBS (50 μL/TA). Myogenic cells were injected into the left TA muscles one day later. First, the myogenic cell cultures were dissociated with 0.05% trypsin–EDTA (Gibco) and resuspended in 10% Matrigel (Corning)/PBS. Under general anesthesia, 60 μL of a cell suspension containing 5 × 10^5^ myogenic cells was injected into the left TA muscle of 6-month-old *NSG-mdx*^4c*v*^ male mice or *NOD/Scid* male mice (3–5 months old) using a 27 G syringe (Terumo). For a negative control, 60 μL of 10% Matrigel/PBS was injected into the right TA muscle. At the same time, 300 μL of a cell suspension containing 1 × 10^6^ MSCs (iMSCs or BM-MSCs) was injected into the intraperitoneal cavity of the mice using a 29 G syringe. For a negative control, PBS vehicle was injected intraperitoneally. The intraperitoneal MSC injections were repeated once per week for the next 3 wks (4 times in total). To examine the effects of uPAR on cell transplantation, Hu5/KD3 cells were cultured for 3 D in 10% FBS/DMEM supplemented with 20 ng/ml recombinant uPAR. Recombinant uPA (1310-SE, R&D Systems) was also added to the culture 24 h before transplantation at a concentration of 20 ng/ml. The cells (1.5 × 10^6^ cells/TA) were then suspended in 50 μl of PBS buffer containing 5% Matrigel and 5 μg/ml uPAR with or without 5 μg/ml uPA and injected into the TA muscles of *NOD/Scid* mice that had been preinjured one day earlier with 1.2% BaCl_2_ solution.

### Histopathological and immunohistochemical analysis

TA muscles and diaphragms were frozen in liquid nitrogen-cooled isopentane, cut into 10-μm sections with a cryostat, and analyzed as previously described [37, 38]. For immunohistochemical staining, the tissue sections were fixed for 10 min in chilled acetone and air-dried. After blocking with 5% goat serum/2% BSA in PBS, the sections were incubated overnight at 4 °C with primary antibodies: anti-human lamin A/C (Santa Cruz), anti-human spectrin (Leica), anti-laminin α 2 chain (clone 4H8-2, Enzo Life Sciences), anti-human lamin B1 (rabbit polyclonal, HistoSure), or anti-dystrophin antibody (rabbit polyclonal, Abcam) (1:100–1:400 dilution). The next day, after washing with PBS, the sections were incubated with fluorescence-labeled secondary antibodies (Alexa 488 goat anti-mouse IgG2b and Alexa568 goat anti-rabbit IgG (Molecular Probes) or Alexa568 goat anti-rat IgG (Molecular Probes)) for 2 h and mounted in Vectashield with DAPI (Vector Laboratories, Inc). Images were recorded with a Keyence microscope BZ-X810. For evaluation of cell engraftment into dystrophin-deficient *mdx* muscles, human lamin A/C (+) dystrophin (+) double-positive myofibers were counted. For CSA measurement, 6 images (200X original magnification) stained with anti-laminin α2 chain antibody from a transverse section of one TA muscle (3 TA muscles/group) were randomly selected. To evaluate fibrosis, the Sirius red-stained area was calculated from three random images/TA (100X original magnification) and analyzed using hybrid cell count software (Keyence).

### Cytokine array

Cytokine levels in culture media were examined using a Human Cytokine Antibody Array (120 targets, Abcam, Cambridge, UK) according to the manufacturers’ instructions. Culture media were harvested from 80–90% confluent cultures of iMSCs derived from 201B7 or 409B2 iPSCs and BM-MSCs. Unused medium (10% FBS/DMEM) was used as a background control. Signals were detected using a ChemiDoc MP Imaging System (Bio-Rad). Data were analyzed with Image Lab 6.0 (Bio-Rad).

### Functional assessment of mdx muscle

The grip strength of mice was measured using a force transducer (Model MK-380 M, Muromachi Kikai). The treadmill running test was performed on a Muromachi Model MK-680 (Muromachi Kikai). After 3 wks of acclimatization, the mice (*n* = 3/group) were subjected to exhaustion tests at 5° inclination with the following protocol: 5 min at 5 m/min followed by incremental speed increases of 1 m/min every 3 min until the mice were exhausted. Exhaustion was defined as the fourth time a mouse spent 5 s on the shocker plate without attempting to re-engage the treadmill.

### Statistical analysis

Data were analyzed and plotted using Prism 8 software (ver. 8.4.0, GraphPad). Means ± SDs or means ± SEMs are shown. Two experimental groups were compared by an unpaired two-tailed Student’s *t*-test. Multiple groups were compared by one-way ANOVA with Sidak’s multiple comparisons test or Dunnett’s test. *, **, ***, and **** indicate *p* < 0.05, *p* < 0.01, *p* < 0.001, and *p* < 0.0001, respectively.

## Supplementary Information

**Additional file 1: Figure S1 (related to Fig. 1)**. No detectable expression of pluripotency factors OCT3/4, NANOG, or SOX2 in iMSCs.** Figure S2 (related to Fig. 2)**. Proliferation of human myogenic cells was stimulated by coculture with iMSCs or BM-MSCs.** Figure S3 (related to Fig. 3)**. Representative immunohistochemistry of cross-sections of the TA muscles of NSG-mdx4Cv mice or NOD/Scid mice transplanted with Hu5/KD3 cells, or Hu5/KD3 cells with iMSCs (i.p.).** Figure S4**. Grip test and treadmill test of mdx mice and Sirius staining of diaphragm in NSG-mdx4cv mice injected intraperitoneally with iMSCs or BM-MSCs.** Figure S5**.BM-MSCs or iMSCs did not affect fiber size in TA muscles of NSG-mdx4Cv or mdx mice.** Figure S6 (related to Table 1)**. iMSCs and BM-MSCs secrete similar but distinct sets of cytokines.** Figure S7 (related to Figure 4)**. Phase contrast images of Hu5/KD3 cells just after scratching with a pipette tip (0 h) and 6 h later (6 h).

## Data Availability

Data set supporting our findings and materials are available from the corresponding author on request.

## Abbreviations

BM: Bone marrow
BDNF: Brain-derived neurotrophic factor
CSA: Cross sectional area
DAPI: 4′,6′-Diamidino-2-phenylindole
DGC: Dystrophin-glycoprotein complex
DMD: Duchenne muscular dystrophy
DMEM: Dulbecco’s modified Eagle’s medium
ECM: Extracellular matrix
FACS: Insulin-like growth factor binding protein 2
FBS: Fetal bovine serum
GAPDH: Glyceraldehyde 3-phosphate dehydrogenase
hiPSCs: Human induced pluripotent stem cells
hESCs: Human embryonic stem cells
iMSC: Induced mesenchymal stem cells
IGF-BP2: Insulin-like growth factor-binding protein 2
IP: Intraperitoneally
IM: Intramuscularly
MTT: [3-(4,5-Dimethylthial-2-yl)-2,5-diphenyltetrazolium bromide
MMP: Matrix metalloproteinase
NOD: Non-obese diabetic
OPG: Osteoprotegerin
PBS: Phosphate-buffered saline
RT-PCR: Reverse transcriptase polymerase chain reaction
SCID: Severe combined immune deficiency
SD: Standard deviation
SEM: Standard error of the mean
TA: Tibialis anterior
uPAR: Urokinase-type plasminogen activator receptor
uPA: Urokinase-type plasminogen activator
