# Supplementary material for: Mesenchymal stem cells derived from human induced pluripotent stem cells improve the engraftment of myogenic cells by secreting urokinase-type plasminogen activator receptor (uPAR)
Source: Stem Cell Res Ther. 2021 Oct 9;12:532. doi: 10.1186/s13287-021-02594-1 (PMC8501581; doi:10.1186/s13287-021-02594-1)
Supplement: Supplementary file 1 — Additional file 1: Figure S1 (related to Fig. 1). No detectable expression of pluripotency factors OCT3/4, NANOG, or SOX2 in iMSCs. Figure S2 (related to Fig. 2). Proliferation of human myogenic cells was stimulated by coculture with iMSCs or BM-MSCs. Figure S3 (related to Fig. 3). Representative immunohistochemistry of cross-sections of the TA muscles of NSG-mdx4Cv mice or NOD/Scid mice transplanted with Hu5/KD3 cells, or Hu5/KD3 cells with iMSCs (i.p.). Figure S4. Grip test and treadmill test of mdx mice and Sirius staining of diaphragm in NSG-mdx4cv mice injected intraperitoneally with iMSCs or BM-MSCs. Figure S5.BM-MSCs or iMSCs did not affect fiber size in TA muscles of NSG-mdx4Cv or mdx mice. Figure S6 (related to Table 1). iMSCs and BM-MSCs secrete similar but distinct sets of cytokines. Figure S7 (related to Figure 4). Phase contrast images of Hu5/KD3 cells just after scratching with a pipette tip (0 h) and 6 h later (6 h). [file 13287_2021_2594_MOESM1_ESM.docx]

**Supplementary Table 1**

**Sequences of qPCR primers used in this study**

| Gene | Forward primer (5’-3’) | Reverse Primer (5’-3’) | Product size (bp) | GenBank No. | Primer Set ID |
| --- | --- | --- | --- | --- | --- |
| ***OCT3/4*** | GACAGGGGGAGGGGAGGAGCTAGG | CTTCCCTCCAACCAGTTGCCCCAAAC | 144 | NM 001173531.2 | Ref. **1, 2** |
| ***NANOG*** | CCTGTGATTTGTGGGCCTGA | CTCTGCAGAAGTGGGTTGTTTG | 168 | NM 001297698.1 | HA237725 (TaKaRa Bio) |
| ***SOX2*** | CCAAGATGCACAACTCGGAGA | CCGGTATTTATAATCCGGGTGCT | 143 | NM 003106.3 | HA173580 (TaKaRa Bio) |
| ***GAPDH*** | GCACCGTCAAGGCTGAGAAC | TGGTGAAGACGCCAGTGGA | 138 | NM 002046.5 | HA067812 (TaKaRa Bio) |

1. Takahashi K, Tanabe K, Ohnuki M, *et al.* ***Cell.*** 2007; 131: 861-872.
2. Okita K, Matsumura Y, Sato Y, *et al.* ***Nat Methods.*** 2011; 8: 409-412.

**Supplementary Figures**

**
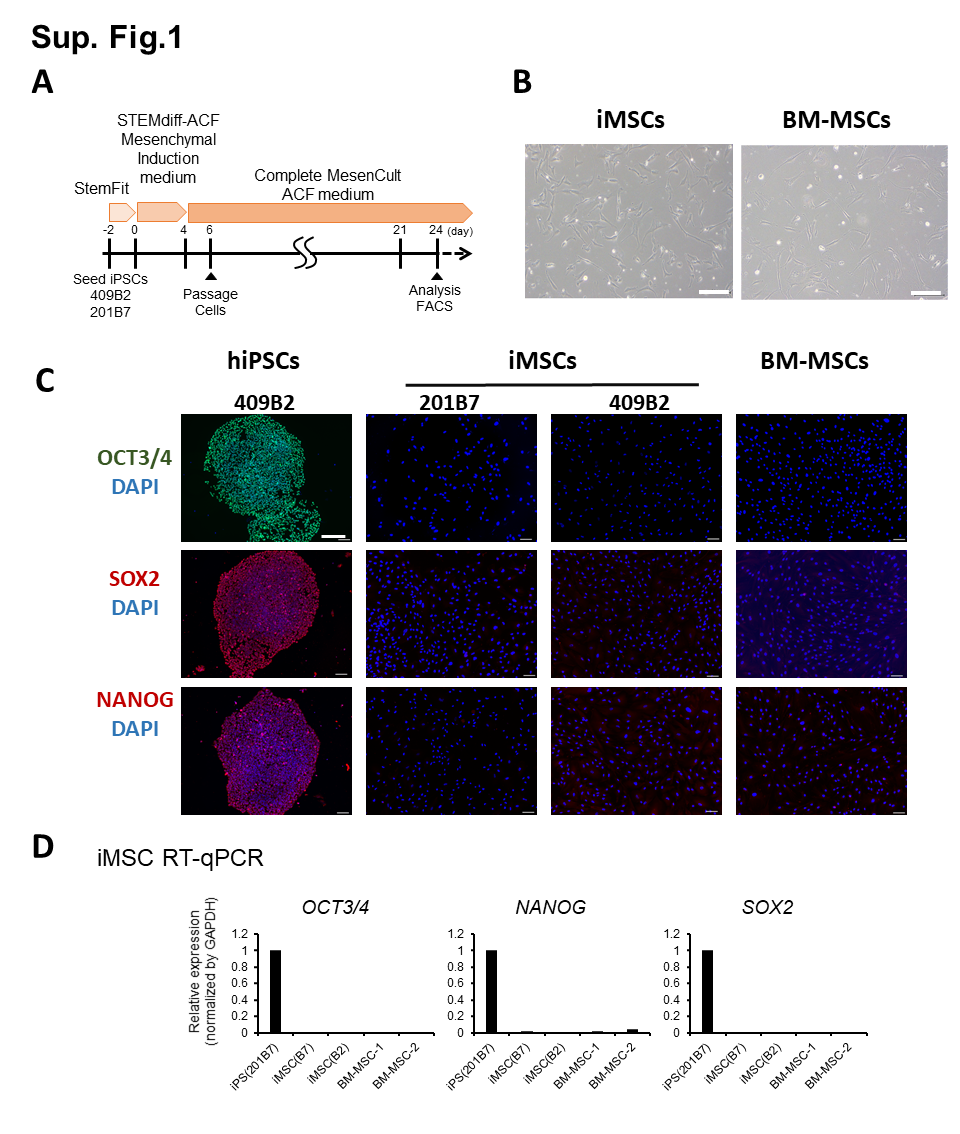
Supplementary Figure 1 (related to Figure 1)**

**No detectable expression of pluripotency factors OCT3/4, NANOG, or SOX2 in iMSCs**

1. Outline of iMSC induction.
2. Phase contrast images of iMSCs and BM-MSCs showing fibroblastic morphology on plastic culture dishes. Scale bar, 100 µm.
3. Representative images of hiPSCs, iMSCs (201B7 hiPSC-derived MSCs and 409B2 hiPSC-derived MSCs), and BM-MSCs immunostained for OCT3/4 (green), SOX2 (red), or NANOG (red) and stained with DAPI (nuclei, blue). Scale bar, 200 μm.
4. RT-qPCR analysis for *OCT3/4,* *NANOG,* and *SOX2* in hiPSCs (201B7), iMSCs (201B7 and 409B2), and BM-MSCs. The signal was normalized to *GAPDH* signal. The analysis was performed three times with consistent results.

**
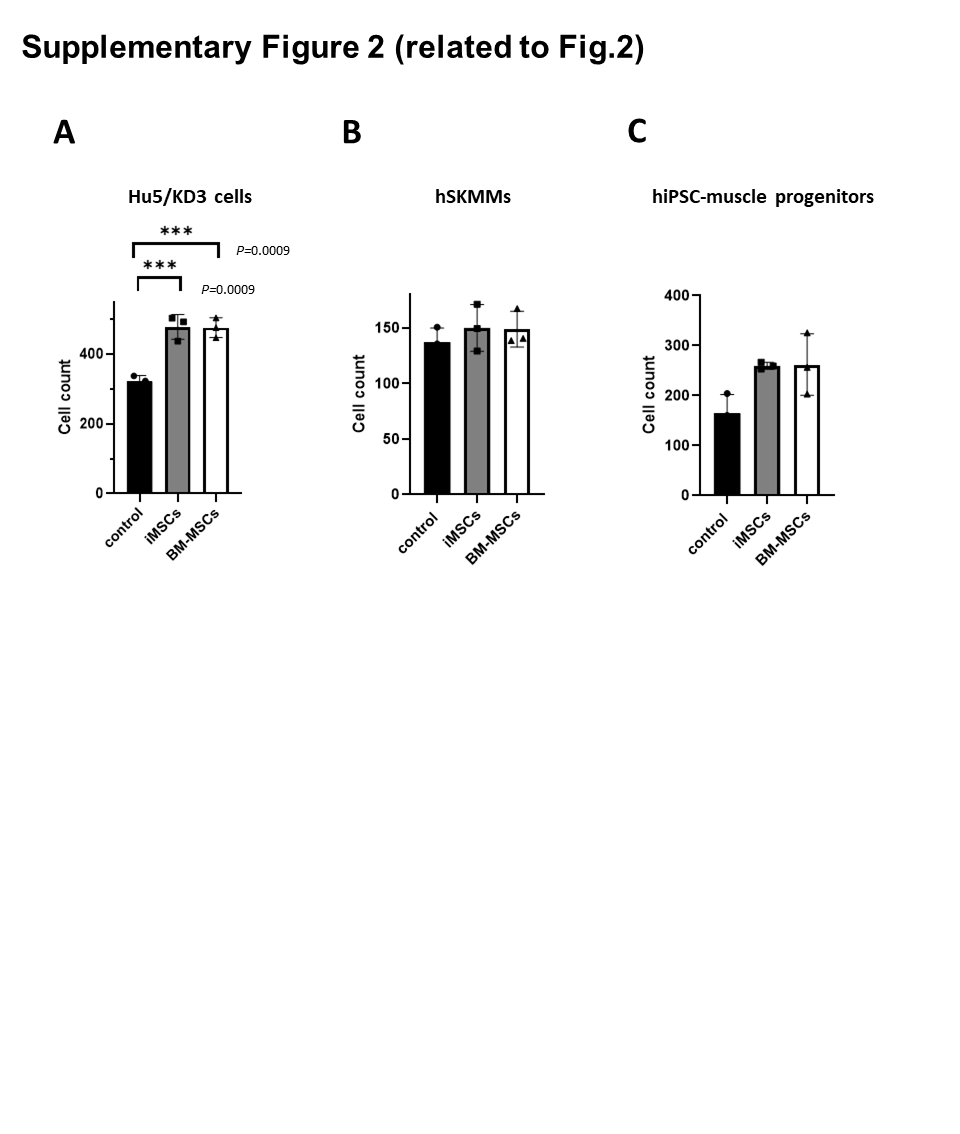
**

**Supplementary Figure 2 (related to Figure 2)**

**Proliferation of human myogenic cells was stimulated by coculture with iMSCs or BM-MSCs**

Hu5/KD3 myogenic cells (**A**), human primary skeletal muscle myoblasts (hSKMM) (**B**), or hiPSC (409B2)-derived muscle progenitors (**C**) were cocultured in a Transwell system with iMSCs (409B7, passage 6) or BM-MSCs (passage 6). After 4 d coculture, the nuclei were stained with DAPI and counted. Means ± SDs. The experiments were performed in triplicate. Thirty microscopic fields were counted for each group. Sidak’s multiple comparisons test.

**
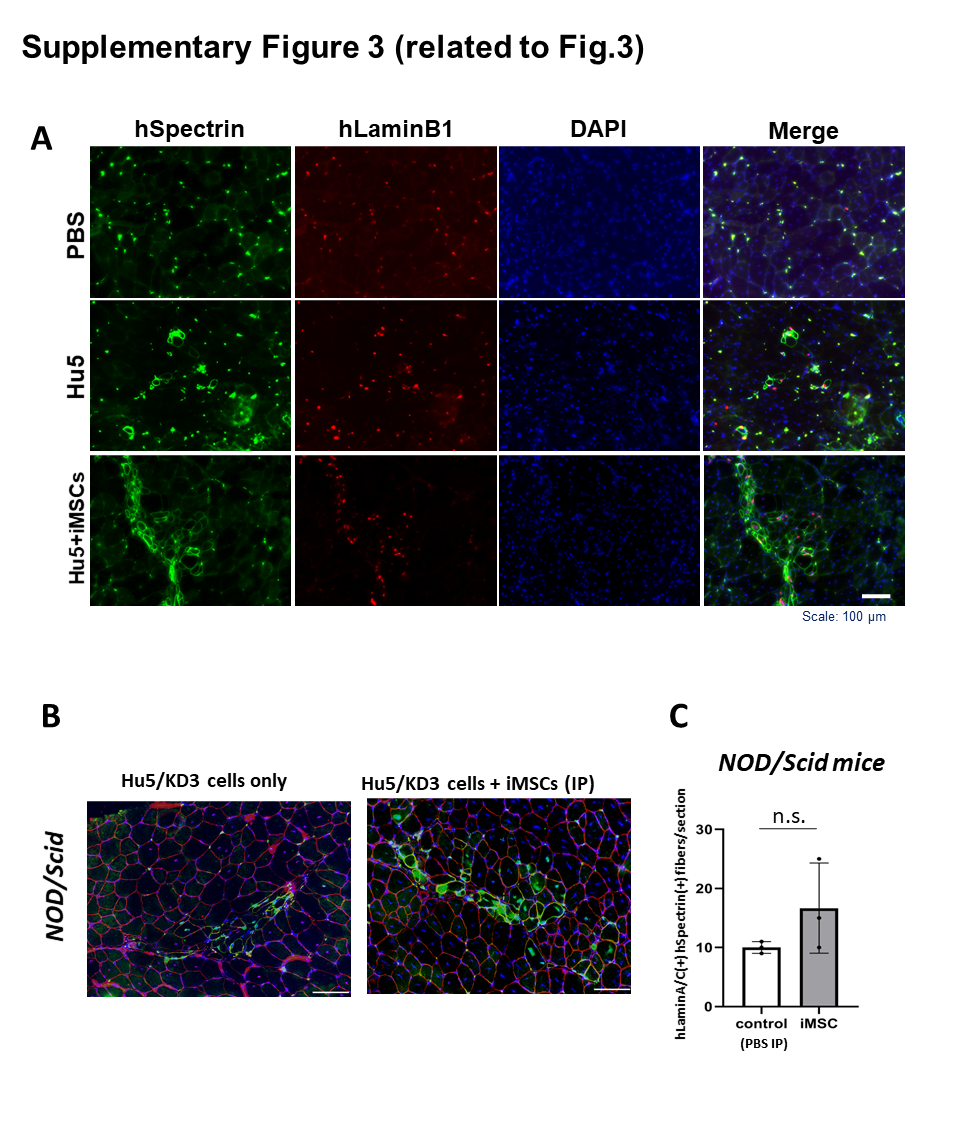
**

**Supplementary Figure 3 (related to Figure 3)**

**iMSCs promote engraftment of human myogenic cells.**

1. Representative immunohistochemistry of cross-sections of the TA muscles of *NSG-mdx^4Cv^ mice* transplanted with PBS, Hu5/KD3 cells, or co-transplantation of Hu5/KD3 cells with iMSCs. The muscle sections were stained with anti-human spectrin (mouse IgG2b; green), anti-human lamin B (rabbit IgG; red), and DAPI (blue). Scale bar, 100 μm.
2. Representative immunohistochemistry of cross-sections of TA muscles of *NOD/Scid mice* transplanted with Hu5/KD3 cells or co-transplanted with iMSCs and Hu5/KD3 cells. The muscle sections were stained with anti-human spectrin (mouse IgG2b; green), anti-human lamin A/C ((mouse IgG2b; green), and anti-laminin alpha2 chain (rat IgG; red) and DAPI (blue). Scale bar, 100 μm.
3. Numbers of human lamin A/C (+) human spectrin (+) fibers/section in the TA muscles of *NOD/Scid* mice directly injected with Hu5/KD3 cells and intraperitoneally injected with iMSCs (iMSCs) or with PBS (control). n=3 mice/group. Student’s t-test. The whole muscle was sectioned, and all images from each sample were analyzed. Shown are the average numbers ± SDs of positive fibers in three consecutive sections in which positive fibers were most frequently found.

**
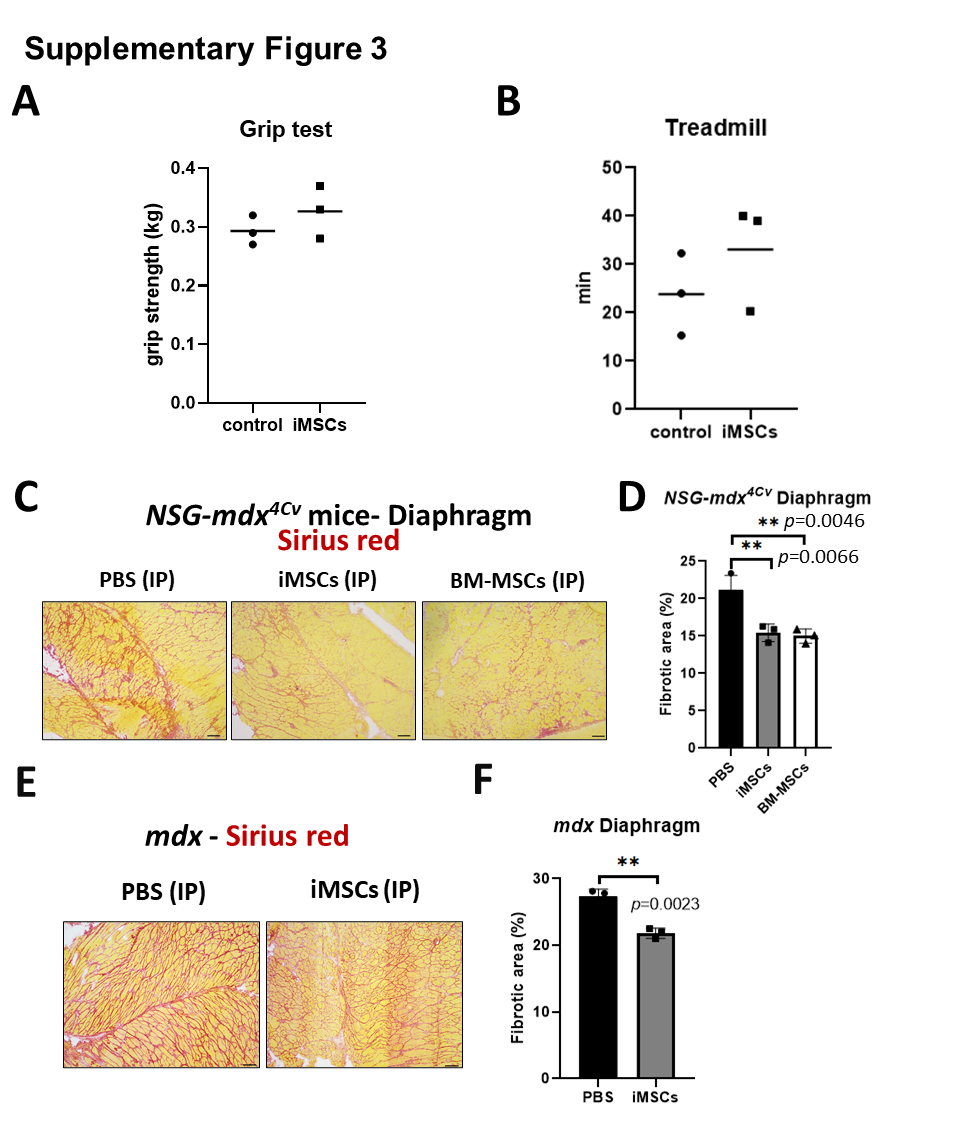
Supplementary Figure 4**

**Grip test and treadmill test of *mdx* mice and Sirius staining of diaphragm in *NSG-mdx^4cv^* mice injected intraperitoneally with iMSCs or BM-MSCs**

1. Grip test of *mdx* mice intraperitoneally injected with PBS (control) or iMSCs performed 4 wk after cell transplantation. n=3 mice/group.
2. A treadmill exhaustion test was performed 4 wk after cell transplantation on *mdx* mice intraperitoneally injected with PBS (control) or iMSCs. n=3 mice/group.
3. Representative Sirius red staining of sections of diaphragms of *NSG-mdx^4Cv^* mice 4 wk after intraperitoneal transplantation (IP) of PBS (control), iMSCs, or BM-MSCs. Scale bar: 100 µm.
4. Percentages of Sirius red-positive areas of the diaphragm of *NSG-mdx^4Cv^* mice. Note that iMSCs and BM-MSCs had significantly reduced fibrotic areas. N=3 mice/group.
5. Representative Sirius red staining of sections of diaphragms of *mdx* mice 4 wk after intraperitoneal transplantation (IP) of PBS (control), or iMSCs. Scale bar: 100 µm.
6. Percentages of Sirius red-positive areas in the diaphragm of *mdx* mice. Note that iMSCs had significantly reduced fibrotic areas. n=3 mice/group. In (D) and (F), three views were taken of each sample. and the mean is shown with the SEM. Sidak’s multiple comparisons test.

**
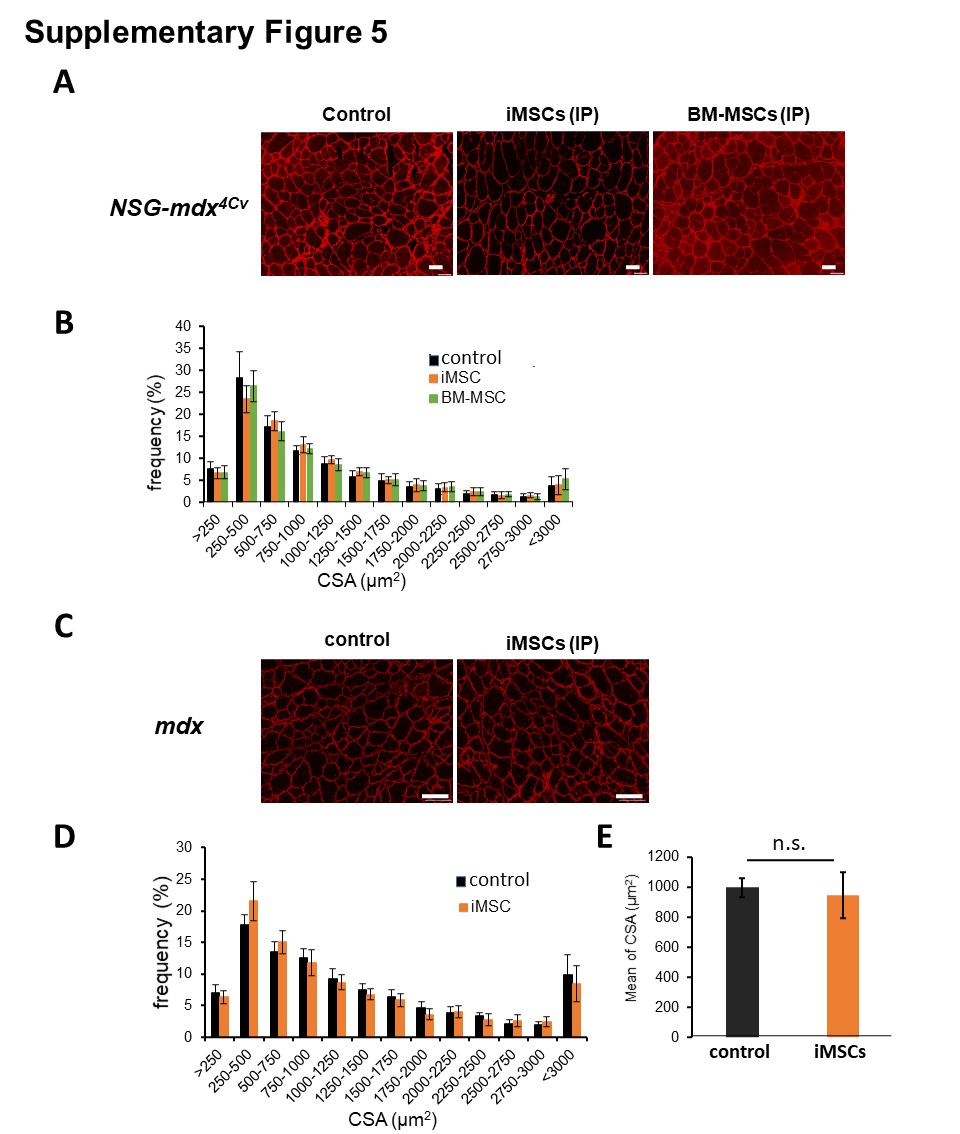
**

**Supplementary Figure 5**

**BM-MSCs or iMSCs did not affect fiber size in TA muscles of *NSG-mdx^4Cv^* or *mdx* mice**

1. Immunostaining of cross-sections of the right TA muscles of *NSG-mdx^4cv^* for laminin α2 chain antibody (4H8-2) after IP injection of PBS (control), iMSCs, or BM-MSCs.
2. Distribution of the cross-sectional areas (CSAs) of myofibers on transverse sections of TA muscles of *NSG-mdx^4Cv^* mice treated with PBS (control), iMSCs or BM-MSCs. Three mice/group. Means ± SDs. The distribution of CSAs did not differ among the three groups. Scale bar, 100 μm.
3. Immunostaining of cross-sections of the right TA muscles of *mdx* mice with a laminin α2 chain antibody (4H8-2) 4 wk after intraperitoneal injection (IP) of PBS (control) or iMSCs. Scale bar, 100 μm.
4. Distribution of CSAs of myofibers in transverse sections of right TA muscles of *mdx* mice 4 wk after IP injection of PBS (control) or iMSCs. There was no significant difference in the distribution of CSAs between the two groups. Three mice/group.
5. Means ± SDs of CSAs in (**D**). n.s., not significant.

**
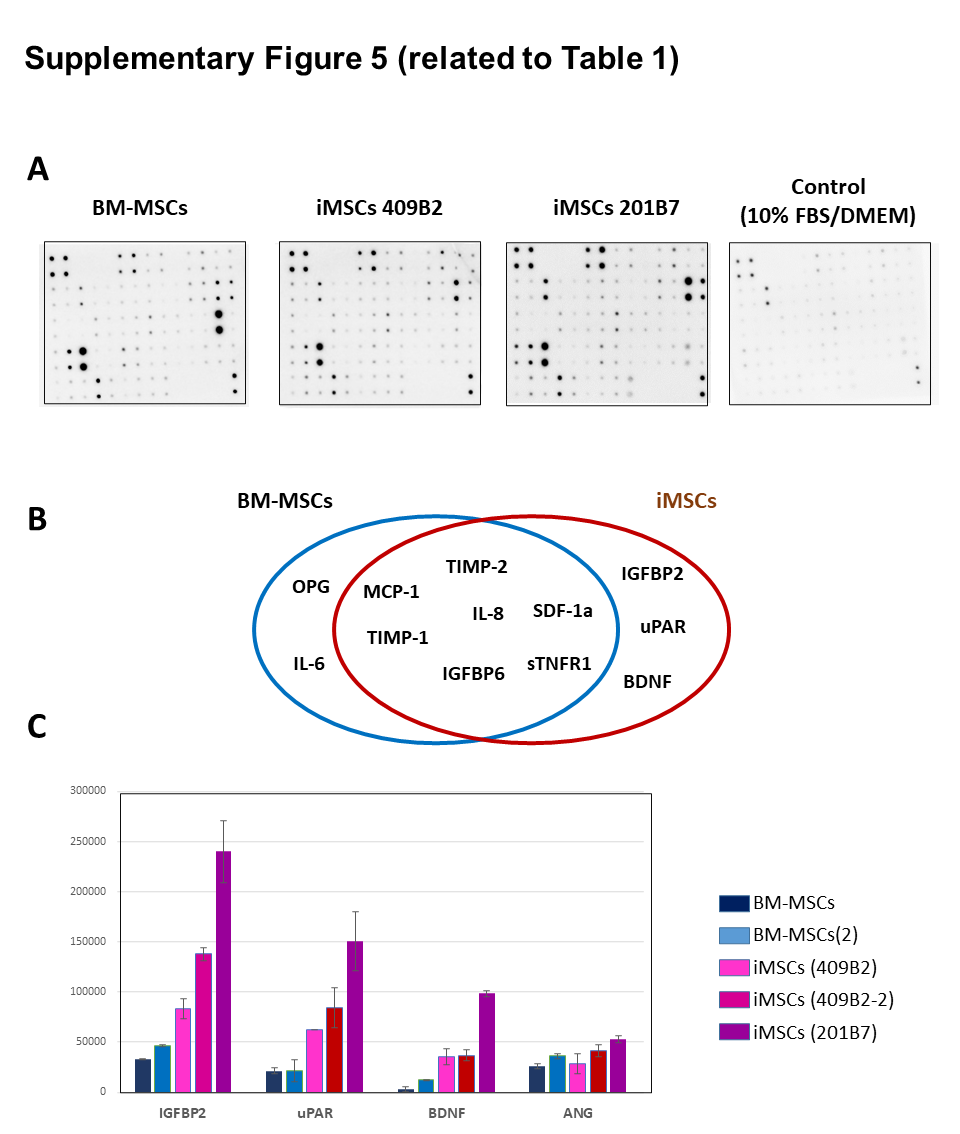
Supplementary Figure 6 (related to Table 1)**

**iMSCs and BM-MSCs secrete similar but distinct sets of cytokines**

1. Representative membrane images of cytokine arrays on a BIO-RAD ChemiDoc MP Imaging System.
2. Venn diagram showing cytokines highly expressed in BM-MSCs (OPG and IL-6) or iMSCs (IGFBP2, uPAR, and BDNF) or expressed at high levels in both BM-MSCs and iMSCs at high levels (TIMP-2, MCP-1, IL-8, TIMP-1, SDF-1a, IGFBP6, and sTNFR1).
3. Relative levels of IGFBP2, uPAR, BDNF, and ANG in the culture medium of BM-MSCs and iMSCs.

**
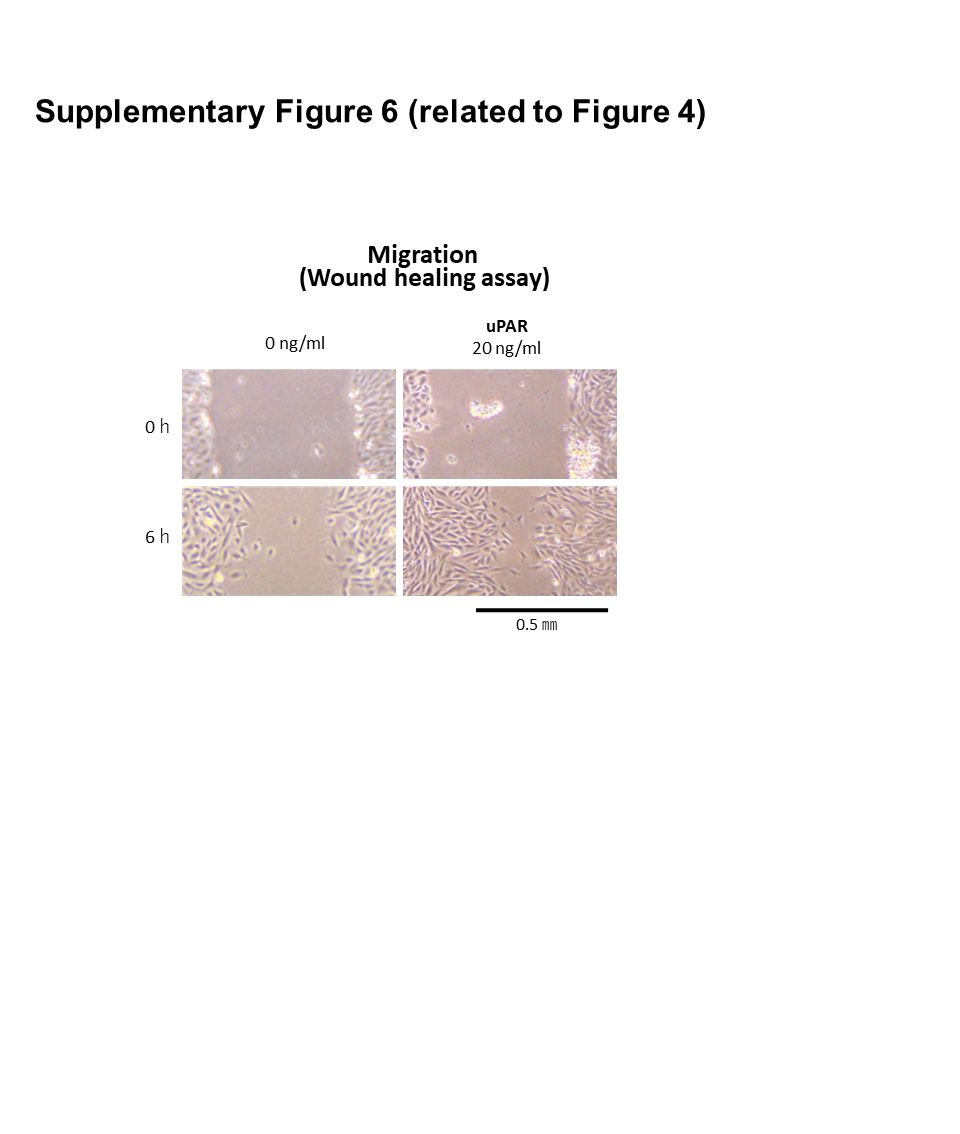
**

**Supplementary Figure 7 (related to Figure 4)**

Phase contrast images of Hu5/KD3 cells just after scratching with a pipette tip (0 h) and 6 h later (6 h). The cells were cultured in 10% FBS/DMEM supplemented with or without 20 ng/ml recombinant uPAR for 24 h before scratching.
